# Phenotype Prediction Using Regularized Regression on Genetic Data in the DREAM5 Systems Genetics B Challenge

**DOI:** 10.1371/journal.pone.0029095

**Published:** 2011-12-28

**Authors:** Po-Ru Loh, George Tucker, Bonnie Berger

**Affiliations:** Department of Mathematics and Computer Science and Artificial Intelligence Laboratory, Massachusetts Institute of Technology, Cambridge, Massachusetts, United States of America; Center for Genomic Regulation, Spain

## Abstract

A major goal of large-scale genomics projects is to enable the use of data from high-throughput experimental methods to predict complex phenotypes such as disease susceptibility. The DREAM5 Systems Genetics B Challenge solicited algorithms to predict soybean plant resistance to the pathogen *Phytophthora sojae* from training sets including phenotype, genotype, and gene expression data. The challenge test set was divided into three subcategories, one requiring prediction based on only genotype data, another on only gene expression data, and the third on both genotype and gene expression data. Here we present our approach, primarily using regularized regression, which received the best-performer award for subchallenge B2 (gene expression only). We found that despite the availability of 941 genotype markers and 28,395 gene expression features, optimal models determined by cross-validation experiments typically used fewer than ten predictors, underscoring the importance of strong regularization in noisy datasets with far more features than samples. We also present substantial analysis of the training and test setup of the challenge, identifying high variance in performance on the gold standard test sets.

## Introduction

Predicting complex phenotypes from genotype or gene expression data is a key step toward personalized medicine: the use of genomic data to improve the health of individuals, for instance by predicting susceptibility to disease or response to treatment [1]–[4]. A pivotal early success in this field was the discovery of gene expression profiles for the classification and prognosis of breast cancer [5]–[7]. Improved technology and declining costs have since enabled ever-larger genetic screens and gene expression studies, allowing researchers to apply the power of genetic analysis of genome-wide gene expression [8], [9]. The difficulty has thus shifted to the algorithmic side: untangling complex associations and identifying small numbers of influential predictors of phenotypic effects amid a sea of largely unrelated measurements [10], [11]. One avenue of recent research has been the integration of distinct types of genomic data to enhance inference, including both linkage studies combining knowledge from different organisms [12], [13] and integrative analysis of distinct data types for the same organism [14], [15].

It is difficult to objectively measure progress on algorithmic challenges without standard benchmarks; within this context, the Dialogue for Reverse Engineering Assessments and Methods (DREAM) initiative [16] aims to provide a fair comparison of methods and a clear sense of the reliability of the models. The fifth annual DREAM challenge held in 2010 included a Systems Genetics component with the goal of predicting disease susceptibility from (1) only genotype data, (2) only gene expression data, and (3) genotype and gene expression data. Through the challenge, the organizers hoped to identify the best predictive modeling approaches and to evaluate the benefits of learning from combined genotype and gene expression data [15].

As a top performer on the second part of the challenge, we were invited to present our results at the DREAM5 conference and contribute to the DREAM5 collection in PLoS ONE; this paper describes our approach. We provide a comparison of several regularized regression models and find comparable performance of elastic net, lasso, and best subset selection. We also carefully analyze the level of noise in the data and consequent variability in performance and offer practical suggestions for similar data analysis and data pre-processing.

## Materials and Methods

### Dataset and challenge setup

The data for this challenge were collected from a systems genetics experiment conducted at the Virginia Bioinformatics Institute [17]. Two inbred lines of soybean plants that differed substantially in susceptibility to a pathogen, *Phytophthora sojae*, were crossed and their offspring were inbred for more than 12 generations to produce a population of recombinant inbred lines (RILs). Individuals within each RIL exhibited almost no genetic variation, whereas distinct RILs displayed much genetic variation owing to their differing mixtures of parental genes. Each RIL was screened for 941 genetic variants and gene-expression profiled for 28,395 genes; gene expression was measured in uninfected plants because the goal of the challenge was to predict disease susceptibility using only information gathered under normal (healthy) conditions.

After infection with *P. sojae*, the plants were assayed for two continuous phenotypes, each a measurement of the amount of pathogen RNA in the infected tissue sample. The first phenotype measured the fraction of pathogen probe sets that yielded a detectable hybridization signal as determined by the MAS5 presence/absence call in the Affymetrix software used to analyze the data. The second phenotype measured the ratio between the sum of all background-subtracted soybean probe intensities and the sum of all background-subtracted pathogen probe intensities. We abbreviate the two phenotypes as P1 and P2.

The training data, from 200 RILs, thus consisted of a 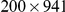 boolean matrix of genotype values (denoting presence or absence of genotype variants), a 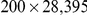 real matrix of gene expression values, and a 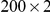 real matrix of phenotype values. Three distinct test sets of 30 RILs each were used for evaluating submissions; 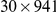 genotype and/or 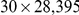 gene expression matrices were provided according to the respective subchallenge conditions, and predictions of the corresponding (withheld) 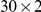 phenotype matrices were solicited. At the end of the submission period, predictions were scored according to their Spearman (rank) correlations to the withheld “gold standard” phenotype data. All training and test data are available at the DREAM5 challenge website (http://wiki.c2b2.columbia.edu/dream/index.php/D5c3).

### Preliminary ranking of predictors by correlation

We began our analysis for this challenge by computing correlation coefficients of the genotype and gene expression training features against the two phenotype variables. The magnitudes of these correlations guided our choice of modeling technique; we also later used correlation-sorted rank lists to limit the scope of computationally intense calculations to those features most likely to be relevant.

On first glance the highest correlations, above 0.3 for the expression data (Table 1), appear promising. The significance of these correlations needs to be considered with the numbers of features in mind, however: 941 genotype and 28,395 gene expression markers. As a rough sanity check, we generated random matrices with sizes equal to those of the training predictor matrices and computed the correlation coefficients of these random features with the training phenotype data. This experiment revealed that in fact the training features as a whole are only very weakly correlated with the phenotypes: almost all correlations from the real training data are within 0.03 of the highest random correlations, and only one real correlation is substantially larger (the 0.34 observed in expression vs. phenotype 2). From the point of view of Bonferroni-corrected p-values, this largest correlation is significant with p-value 0.017; all other p-values exceed 0.1 upon applying the Benjamini-Hochberg multiple hypothesis correction [18].

**Table 1:** Highest absolute correlations of genotype and gene expression data to phenotype, versus random background.

Top correlations	Genotype		Expression	
(absolute values)	Training	Random	Training	Random
Phenotype 1	0.2155	0.2404	0.3034	0.2835
	0.2122	0.2116	0.2976	0.2781
	0.2061	0.1862	0.2975	0.2749
	0.2054	0.1857	0.2963	0.2689
	0.2041	0.1851	0.2909	0.2611
Phenotype 2	0.2433	0.2127	0.3441	0.2777
	0.2261	0.2104	0.3084	0.2684
	0.2198	0.2053	0.2990	0.2679
	0.2181	0.1928	0.2824	0.2642
	0.2180	0.1926	0.2754	0.2619

The top five correlations found in the training data are shown, as are the top five correlations against a random 0–1 matrix with the same dimensions as the genotype data and a random normal matrix replacing the gene expression data.

These observations suggest that most features have little or no predictive power, and hence proper regularization is crucial for modeling this dataset. Additionally, the small difference between training correlations and the random background distribution indicate that the prediction task at hand is difficult; the amount of signal in the data is likely quite small.

In light of the above considerations, we sought to keep our modeling simple and chose regularized regression as our general approach. Before fitting the data, however, we needed to ensure that the relation between predictor and response variables was as linear as possible, and so we considered data transformations and basis expansions.

### Rank transformation to reduce phenotype outliers

**Figure 1:**
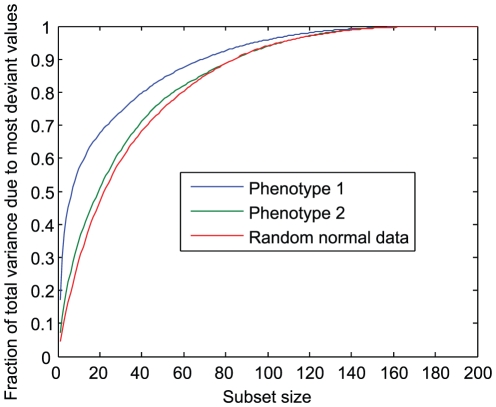
Large contribution of outliers to variance in phenotype 1. The largest seven outliers in phenotype 1 account for the bulk of the variance in the data; in contrast, the outlier distribution for phenotype 2 is similar to that of a random normal variable.

Upon plotting the phenotype training data, we discovered that the variance in the distribution of phenotype 1 is dominated by outliers. Among the 200 measurements of phenotype 1, the largest outlier is 5.83 sample standard deviations from the mean. Moreover, the seven most deviant samples account for more than half of the total variance. For phenotype 2, the largest outlier is a substantial 3.77 standard deviations above the mean but overall the distribution does not have unusually long tails compared to a normal distribution. A plot of the fractions of variance explained by increasing subsets of largest outliers in phenotype 1, phenotype 2, and random data illustrates this behavior (Figure 1).

Motivated by the Spearman correlation-based scoring scheme used in this challenge, which judges predictions based on ordering rather than absolute accuracy, we applied a rank transformation to phenotype 1 to remove the impact of outliers on regression models. More precisely, we replaced the numerical values of phenotype 1 measurements with their ranks among the 200 sorted samples. Because the approaches we applied minimized squared error (along with regularization terms), asking our models to predict ranks rather than actual values removed the heavy weight that outlier values would otherwise have received. Absolute predictions could of course be recovered by interpolation if desired.

### Basis expansion to boolean combinations of genotype variables

With only binary genotype data available for prediction in subchallenge B1, we hypothesized that the true phenotypic response for a genotyped sample would be far from linear. The simplest possible example of a nonlinear effect is interaction between genotype markers: for instance, if two genes act as substitutes for one another, their function is only suppressed if both are turned off. Similarly, if two genes are critical to different parts of a pathway, turning off either one would impair its function.

With these examples in mind, we considered applying logic regression [19] to expand the set of features available to our linear models to include boolean combinations of each pair 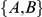 of genotype features:
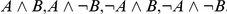
Note that the complements of these relations are implicitly included by a linear model as well, so together they cover all nontrivial binary boolean relations.

**Figure 2:**
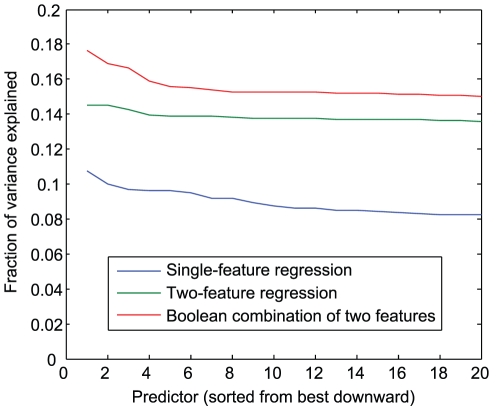
Single-variable, two-variable, and pairwise logic regression for phenotype 2. The plot compares the best least squares fits attainable under three model types: single-variable regression using each genotype feature independently (blue), two-variable regression using pairs of features at once (green), and single-variable regression using pairs of features combined through a binary boolean relation (red). The best single-variable fits using boolean combination features outperform the best two-variable regressions.

To gauge the efficacy of these combined features, we compared the largest fractions of variance explained by single boolean combination features (using single-variable least-squares regression) to the best fits obtained by two-variable regression on pairs of the original genotype features. Looking at the 20 best-performing regressions from each group (Figure 2), we see that the top boolean combinations outperform the best two-variable regressor pairs, suggesting that basis expansion in this manner does indeed improve our ability to fit the data.

An important caveat to keep in mind when interpreting these measurements is that the number of feature combinations considered is very large (nearly 2 million), thus allowing random chance to inflate best performances as in the case of correlations examined above. Nonetheless, we expect that the relative trends are still informative.

**Figure 3:**
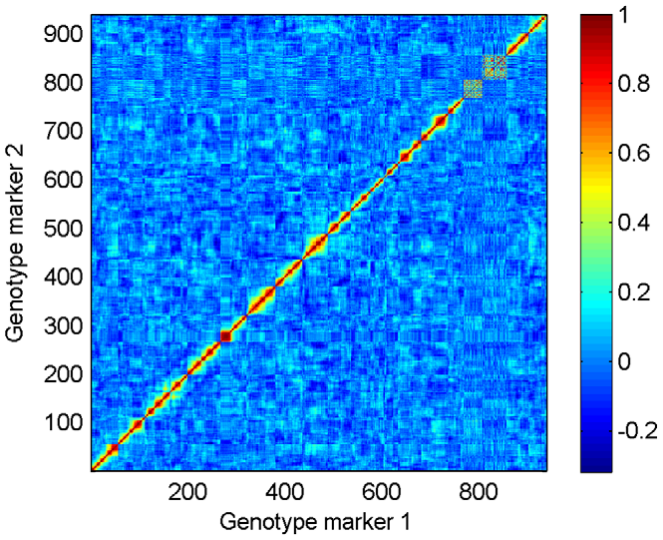
Correlation coefficients between genotype markers, displaying linkage disequilibrium. The heat map shows Pearson correlations between pairs of genotype markers; most pairs have only slightly positive or negative correlations attributable to chance, but groups of nearby markers exhibit distinctly positive correlations.

Upon closer inspection of the best boolean combination markers, we discovered that some were near-trivial due to linkage disequilibrium (Figure 3): for instance, we observed cases of nearby markers 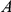 and 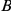 having identical values for 198 out of 200 samples, so that the boolean combination 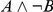 was nonzero for only two samples. Such combinations are very noisy (and likely uninformative) predictors; we therefore limited the boolean features under consideration to those containing at least 20 nonzeros.

### Regularized regression modeling

Having taken steps to linearize the predictor-response relationship, we applied regularized regression to model the data. Classical linear regression on a predictor matrix 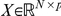 and response vector 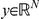 assumes a model 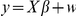 (where 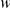 represents noise) and finds the coefficient vector 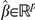 minimizing the sum of squared residuals 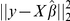. In the highly underconstrained case (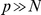), however, additional constraints must be imposed for there to be any hope of approximating 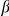; often one assumes that 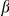 is sparse, in which case 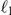-minimization techniques may be applied [20]. In the context of our experimental setup this assumption means that most genetic markers and expression values are unrelated to phenotype, which seems reasonable.

Our main approach of choice was elastic net regression [21], which imposes constraints on model complexity by adding the following penalization term to the squared residuals being minimized:
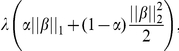
where 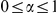 determines the weighting of the two terms and 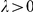 is the strength of the regularization. Note that 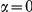 produces the ridge regression penalty while 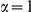 gives the lasso; thus, in some sense elastic nets interpolate between 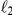
- and 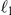-regularization. Elastic net regression can be computed efficiently; we used the glmnet package available for Matlab [22].

For the purpose of comparison, we also tried fitting the data with a simple best subset selection approach, which seeks to minimize squared error using only a limited number of regressors. (In the language of our above discussion, this constraint can equivalently be viewed as imposing an 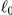 penalty 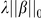.) Because best subset selection is a nonconvex combinatorial problem with exponential complexity, however, finding best subsets exactly was computationally intractable [23]; instead, we performed simulated annealing on a subset of likely candidate features (chosen by correlation-ranking within our cross-validation loop) to obtain a reasonable approximation.

Implementation details are as follows. For elastic net regression, we ran glmnet with 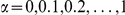 and uniformly log-spaced regularization path and default values of all other parameters. The best pair of 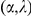 for the elastic net was then selected to achieve optimal cross-validation performance. For the lasso, we ran glmnet with 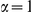 and default values of all other parameters. In this case, glmnet automatically calculated a regularization path and we selected the least complex model achieving within one standard deviation of the best cross-validation performance. We used this value of 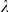 for our final regression fit.

For best subset selection, we first filtered to the top 30 features with strongest correlations to phenotype (recomputed for each cross-validation training set). We then used simulated annealing to compute subsets of size 1–20 features obtaining approximately optimal linear fits to each training fold. The annealing procedure consisted of 5 runs of initialization with a random feature subset of the required size followed by 5000 iterations of attempted swaps, using a linear cooling schedule. Explictly, the acceptance probability of a swap was 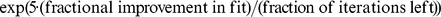, capped at 1.

## Results

### Modest performance of all regression techniques on training dataset

We evaluated our regression methods using 7-fold cross-validation on the 200-sample training set, measuring goodness of fit with Spearman correlation to match the DREAM evaluation criterion. We chose to use 7 folds so that our cross-validation test sets during development would have approximately the same size as the 30-sample gold standard validation set, allowing us to also estimate the performance variance to be expected on the validation set. We applied each regression technique–elastic net, lasso, and approximate best subset selection with simulated annealing–to fit phenotype 1 (rank-transformed) and phenotype 2 individually, using sets of regressors corresponding to the three subchallenges of DREAM5 Systems Genetics B: genotype only (B1), gene expression only (B2), and both genotype and expression (B3). Within subchallenge B1, we ran two sets of model fits, one using only raw genotype markers as regressors and the other using the boolean basis expansion described in Methods.

**Figure 4:**
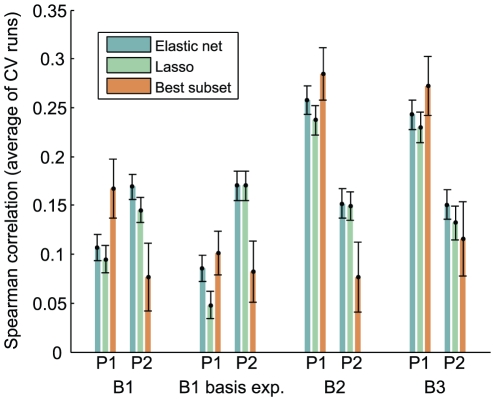
Goodness of fit of regularized regression models on training data using various regressor sets. We tested elastic net, lasso, and approximate best subset selection on phenotypes 1 and 2 using regressor sets derived from the DREAM5 subchallenges B1, B2, and B3. In each case the regularization parameter(s) were chosen to optimize average Spearman correlation. We ran multiple cross-validation tests with different random fold splits to reduce uncertainty in mean performance and enable comparison between methods; error bars show one standard deviation of confidence.

Because of the relatively small number of samples and large number of predictors, the random assignment of samples to cross-validation folds caused substantial fluctuation in performance, even when averaging across folds. We overcame this difficulty by running multiple cross-validation tests for each model fit using different fold assignments in each run (20 replicates for elastic net and lasso and 5 replicates for best subset selection), thus obtaining both mean performances and estimates of uncertainty in each mean. We chose regularization parameters for each method in each situation to optimize mean performance; Figure 4 shows the results using these parameters.

Overall, the three regularized regression techniques perform quite comparably. Note that elastic net regression necessarily always performs at least as well as lasso (because lasso corresponds to the elastic net with parameter choice 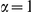); however, the performance difference is very small in all cases. Best subset selection appears to perform slightly better than the others in predicting phenotype 1 and somewhat worse in predicting phenotype 2.

Comparing the different regressor sets, subchallenge B1 with genotype data only is clearly the most difficult. The availability of gene expression data in subchallenges B2 and B3 dramatically boosts average Spearman correlations to the 0.25–0.3 range for phenotype 1 (though performance for phenotype 2 is largely unchanged in the 0.15–0.2 range typical for all other cases). Unfortunately, our regression models did not attain a performance increase from B2 to B3 with the inclusion of genotype data along with expression data, nor did boolean basis expansion appear to help with performance on B1.

### Effectiveness of rank transformation on phenotype 1

**Figure 5:**
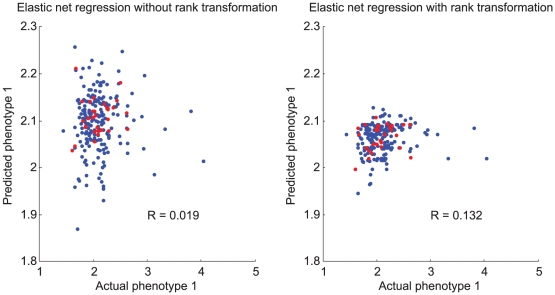
Example elastic net predictions versus actual values with and without rank transformation for subchallenge B2P1. Each scatter plot shows predictions from one cross-validation run on the training data (blue points) as well as predictions of the fitted model for the gold standard test set (red points). For the elastic net modeling on rank-transformed data (right plot), predictions of phenotype 1 values on an absolute scale were obtained by interpolation. The reported values of 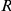 are the Pearson correlation coefficients.

Surprisingly, the rank transformation we applied to phenotype 1 turned out to have the greatest impact of the pre-regression data transformations we attempted. For the purpose of comparison, we performed the same model-fitting as above using raw (untransformed) values of phenotype 1. In all cases the rank transformation increases average Spearman correlations considerably (Table 2). For subchallenges B2 and B3, rank-transforming phenotype 1 more than doubles the correlation that would otherwise be achieved, though a look at scatter plots of predicted versus actual values (Figure 5) shows that our predictive power is still marginal: predictions are compressed toward the mean, as tends to occur when trying to apply regression to data that is difficult to model. The effectiveness of the rank transformation was unique to phenotype 1; in contrast, rank-transforming phenotype 2 had no significant effect.

**Table 2:** Improvement in goodness of fit with rank transformation on phenotype 1.

	Spearman corr. before and after transformation					
Subchallenge (regressors)	Elastic net		Lasso		Best subset	
B1 (genotype)	0.058	0.107	0.054	0.095	0.092	0.167
B1 (genotype with basis expansion)	0.042	0.085	0.011	0.048	0.025	0.102
B2 (expression)	0.099	0.257	0.094	0.237	0.111	0.285
B3 (genotype and expression)	0.090	0.243	0.077	0.230	0.092	0.272

Applying the rank transform to phenotype 1 increases average cross-validated Spearman correlations for all regression approaches and regressor sets we tested. The performance improvement is especially large for subchallenges B2 and B3, where gene expression data is available.

### Strong regularization in best-fit models

**Figure 6:**
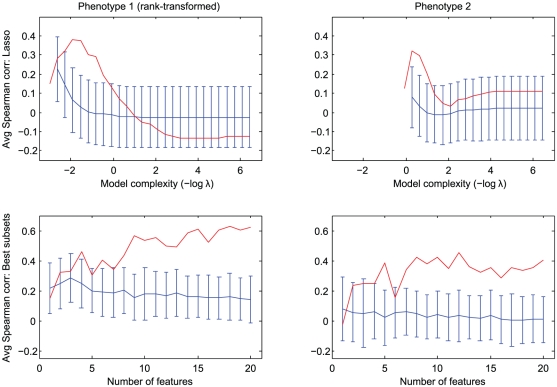
Variation in cross-validation and test set performance with model complexity for subchallenge B2. Each plot follows the performance of a regression model as complexity increases. For lasso (top plots), model complexity is determined by a regularization parameter 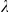; for best subset selection (bottom plots), complexity is defined as the number of features used. The blue curves show Spearman correlations averaged over cross-validation folds, each fold having approximately the same size as the gold standard test set. Performance varies dramatically from fold to fold; error bars show one standard deviation of the Spearman correlations achieved for different folds. The red curves follow performance of the models on the actual gold standard.

Taking a closer look at the optimal regularization parameters for elastic net, lasso, and approximate best subset selection, we discovered strikingly low model complexity prescribed by cross-validation in each case. As an example, the blue curves of Figure 6 plot average performance of lasso and best subset selection on subchallenge B2 as a function of increasing model complexity. (Note that unlike typical cross-validation curves with error to be minimized on the vertical axis, our performance metric is Spearman correlation so we seek maxima.) The regularization parameter is particularly transparent for best subset selection (shown in the bottom two plots): in this case, regularization is explicitly manifested as the number of features to be used in the subset chosen for regression.

With lasso, we likewise see that performance drops off quickly as model complexity increases; here, the complexity parameter 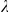 is less directly interpretable, but since the 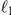-minimization approach of lasso also results in sparse models, the result in this case as well is that lasso also recommends using only a handful of features. Even with elastic net regression, which tends to fit denser models due to the presence of an 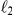 “ridge” penalty, we find that optimal regularization parameter choices de-emphasize the ridge term, creating lasso-like model fits with 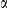 (the “lasso proportion”) typically in the range 0.8 to 1.

**Figure 7:**
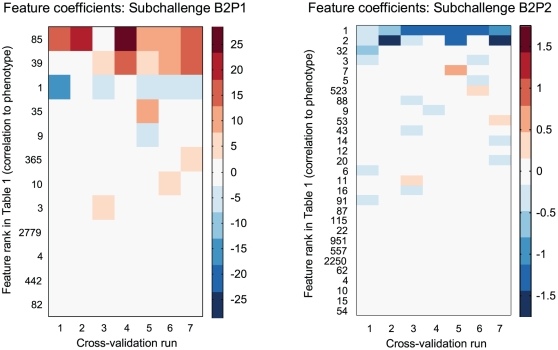
Stability of features and coefficients selected by elastic net regression for subchallenge B2. The heat maps show regression coefficients chosen by the best-fit elastic net models as each cross-validation fold is in turn held out of the training set. The features shown on the vertical axis are those having a nonzero coefficient in at least one of the seven runs; they are indexed by their rank in Table 1, correlation to the phenotype being predicted.

To better understand the strong regularization, we provide heat maps displaying the feature weight distributions chosen by the elastic net to predict phenotype 1 (rank-transformed) and phenotype 2 for a set of cross-validation runs on subchallenge B2 (Figure 7). As expected, the few features chosen from the 28,395 available are typically among those predicted to be most informative according to correlation with phenotype (Table 1). The features assigned greatest weight are quite stable from fold to fold, while the choice of lower-weight features is noisier.

### High variance in performance on individual cross-validation folds and test set

As mentioned earlier, our cross-validation analysis also allows us to estimate the accuracy to which algorithm performance can be measured using a 30-sample test set. Unfortunately, we find that this test size is insufficient for accurate evaluation: whereas the greatest-weight features selected by our models are relatively stable from fold to fold (Figure 7), the Spearman correlations obtained on the held-out test folds vary markedly. The blue error bars in Figure 6 display one standard deviation in the Spearman correlation between predicted and actual phenotype values from fold to fold; with 7-fold cross validation, each fold contains about 29 samples. These standard deviations mostly fall in the 0.15–0.2 range, in some cases exceeding the mean performance of even the best parameter choice.

The red curves of Figure 6 illustrate the variance in performance when models fit on the training data were applied to the actual 30-sample gold standard test set (released after the end of the DREAM5 challenge). As expected, test set performance strays substantially from the mean.

### Official DREAM5 challenge results

**Figure 8:**
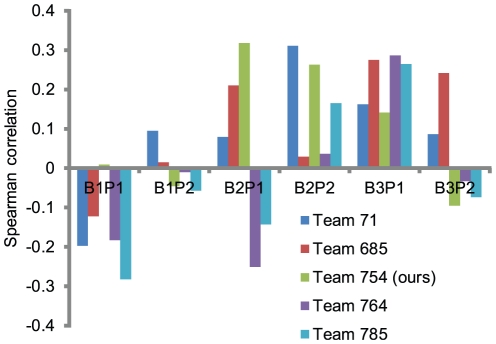
Final results of the five teams participating in all three DREAM5 Systems Genetics B subchallenges. All teams had difficulty even achieving consistently positive correlations; we suspect the main obstacles were the large amount of noise in the data and the small 30-sample gold standard evaluation sets. We achieved the best performance on the test set used for subchallenge B2 (prediction using gene expression data only).

Notwithstanding the caveat just discussed regarding uncertainty in results on a small test size, we include the final results from the DREAM5 Systems Genetics B challenge for completeness (Figure 8). Our team, identified by “orangeballs” and Team 754 in the published results, achieved the best performance on the subchallenge B2 test set. The overall distribution of Spearman correlations achieved by the various teams is in line with what we would expect given our analysis of our training results, with subchallenges B2 and B3 being more tractable than B1.

## Discussion

While the performance achieved by our methods–indeed, by every team's methods–is modest, our work does highlight a few important lessons in statistical learning and in the setup of algorithmic benchmarking challenges such as DREAM. Regarding the first, our analysis did not lead us to a radically new and complex model for the genotype-phenotype relationship in *P. sojae*; on the contrary, we found that given the limitations of small sample size and noise in the training data, the best models we discovered were among the simplest we tried. Regularized least squares regression with careful cross-validation and linearization (using the rank transform we applied to phenotype 1) proved to be as effective an approach as any other we are aware of, and the noise-to-signal in the data was such that the best linear fits needed only a few well-chosen regressors.

One might hope that the transparency of such simple models can shed light on the underlying biological mechanism at work; while this may be possible, we also should caution against trying to glean more from the models than the data allow. Simplicity may be due to the involvement of only relatively few genes or just to the fact that heavy regularization makes models less prone to overfitting. In light of the noisiness of the dataset, we suspect the latter may be true. As a case in point, while we were disappointed that modeling pairwise interactions through boolean basis expansion did not improve fitting using the genotype data, we still find it quite plausible that such effects are at work and may aid modeling in situations when more data is available. With this dataset, our techniques were likely unable to discern these effects because the limited data size could not support the increased complexity that modeling interactions would entail.

Overall, while this contest was perhaps too ambitious for the data available, we feel it succeeded in stimulating research and discussion in the field. The original motivation of developing methodology for combining genotype and gene expression data to improve phenotype prediction remains a worthy goal and interesting open question.
